# Tools for genetic manipulation of the plant growth-promoting bacterium *Azospirillum amazonense*

**DOI:** 10.1186/1471-2180-11-107

**Published:** 2011-05-16

**Authors:** Fernando H Sant'Anna, Dieime S Andrade, Débora B Trentini, Shana S Weber, Irene S Schrank

**Affiliations:** 1 Centro de Biotecnologia, Universidade Federal do Rio Grande do Sul (Av. Bento Gonçalves, 9500, Campus do Vale), Porto Alegre, RS, Brazil; 2 Departamento de Biologia Molecular e Biotecnologia - Centro de Biotecnologia, Universidade Federal do Rio Grande do Sul (Av. Bento Gonçalves, 9500, Campus do Vale), RS, Brazil

## Abstract

**Background:**

*Azospirillum amazonense *has potential to be used as agricultural inoculant since it promotes plant growth without causing pollution, unlike industrial fertilizers. Owing to this fact, the study of this species has gained interest. However, a detailed understanding of its genetics and physiology is limited by the absence of appropriate genetic tools for the study of this species.

**Results:**

Conjugation and electrotransformation methods were established utilizing vectors with broad host-replication origins (pVS1 and pBBR1). Two genes of interest - *glnK *and *glnB*, encoding PII regulatory proteins - were isolated. Furthermore, *glnK*-specific *A. amazonense *mutants were generated utilizing the pK19MOBSACB vector system. Finally, a promoter analysis protocol based on fluorescent protein expression was optimized to aid genetic regulation studies on this bacterium.

**Conclusion:**

In this work, genetic tools that can support the study of *A. amazonense *were described. These methods could provide a better understanding of the genetic mechanisms of this species that underlie its plant growth promotion.

## Background

Many of the negative ecological impacts of agriculture originate from the high input of fertilizers. The increase of crop production in the future raises concerns about how to establish sustainable agriculture; that is, agricultural practices that are less adverse to the surrounding environment [1,2]. The use of microorganisms capable of increasing harvests is an ecologically compatible strategy as it could reduce the utilization of industrial fertilizers and, therefore, their pollutant outcomes [1,3].

*Azospirillum *is a well-known genus that includes bacterial species that can promote plant growth. This remarkable characteristic is attributed to a combination of mechanisms, including the biosynthesis of phytohormones and the fixation of nitrogen, the most intensively studied abilities of these bacteria [4]. The species *Azospirillum amazonense *was isolated from forage grasses and plants belonging to the Palmaceae family in Brazil by Magalhães et al. (1983) [5], and subsequent works demonstrated its association with rice, sorghum, maize, sugarcane, and *Brachiaria*, mainly in tropical countries [6]. When compared with *Azospirillum brasilense*, the most frequently studied species of the genus, *A. amazonense *has prominent characteristics such as its ability to fix nitrogen when in the presence of nitrogen [7] and its better adaptations to acidic soil, the predominant soil type in Brazil [5,8]. Moreover, Rodrigues et al. (2008) [8] reported that the plant growth promotion effect of *A. amazonense *on rice plants grown under greenhouse conditions is mainly due to its biological nitrogen fixation contribution, in contrast to the hormonal effect observed in the other *Azospirillum *species studied.

Despite the potential use of *A. amazonense *as an agricultural inoculant, there is scarce knowledge of its genetics and, consequently, its physiology. Currently, the genome of *A. amazonense *is being analyzed by our group and its completion will be forthcoming; therefore, the development of specific genetic tools is crucial for taking full benefit of the data. that will be generated. Hence, in this work we describe methods for the genetic manipulation of *A. amazonense*: DNA transfer methodologies (conjugation and electroporation), reporter vectors, and site-directed mutagenesis. In order to demonstrate the applicability of the optimized techniques, we show the results obtained in the study of the PII signaling proteins of *A. amazonense*, starting from their gene isolation.

## Results and Discussion

### Isolation of *glnB *and *glnK *genes from *A. amazonense*

The PII proteins are pivotal regulators of the nitrogen metabolism, controlling the activities of transporters, enzymes and transcriptional factors implicated in this process [9,10]. These proteins are highly conserved and are widely distributed throughout prokaryotes [11]. In Proteobacteria in particular, there are two main types of PII proteins, GlnB and GlnK. In this work, two PII protein encoding genes from *A. amazonense *were isolated. Southern blot analysis utilizing a PCR-generated *glnB *fragment as the probe revealed two distinct signals in the genomic DNA of *A. amazonense *digested with SalI: the strongest at the ~2 kb DNA fragments and the weakest at the ~3 kb DNA fragments (data not shown). Based on these results, a genomic library enriched with 2-3 kb SalI fragments was constructed. The library was partially sequenced and a PII protein homolog was identified. The deduced amino acid sequence of this gene was found to be highly similar to that of the GlnZ proteins (GlnK-like homologs) from *A. brasilense *and *Azospirillum *sp. B510 (75% identity and 86% similarity), and *Rhodospirillum. centenum *(73% identity and 86% similarity). Arcondéguy et al. (2001) [12] suggested that the *glnZ *genes should be termed *glnK*, since their deduced proteins are highly similar to the GlnK proteins. Furthermore, there is a functional correspondence between these proteins, as both regulate the uptake of ammonium through the AmtB transporters [13-15]. Therefore, we adopted the *glnK *designation for this *A. amazonense *homolog, mainly because this nomenclature could facilitate comparisons between other bacterial systems.

**Figure 1:**
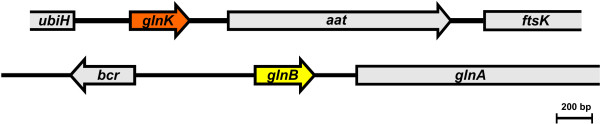
**Physical maps of the *glnK *and *glnB *regions of *A. Amazonense***. Genes are represented by the large arrows; *glnA*, *ubiH *and *ftsK *were not completely sequenced.

The *glnK *gene from *A. amazonense *is flanked by the *aat *gene in the downstream region, which codes a putative aspartate aminotransferase and the *ubiH *gene in the upstream region, which codes an enzyme implicated in ubiquinone biosynthesis (Figure 1). This genetic organization resembles that found in other species from the Rhodospirillales order, namely *A. brasilense*, *Azospirillum *sp. B510 and *R. centenum*.

Since the *glnB *gene was not found in the genomic library, the Inverse PCR methodology was carried out to isolate this gene. A ~2 kb amplicon that contained the *glnB *gene was obtained (data not shown). It was found that the protein of this gene displays 92% identity and 98% similarity to the GlnB proteins from *Azospirillum *sp. B510 and *A. brasilense*, and 96% identity and 98% similarity to the GlnB protein of *R. centenum*. The *glnB *gene is located upstream of the *glnA *gene (glutamine synthetase), the same genetic context observed in these bacteria (Figure 1).

In *A. brasilense*, *glnB *has a key role in nitrogen fixation because its protein product regulates the activity of NifA, the transcriptional factor of nitrogen fixation [16,17].

Furthermore, both of the GlnZ (GlnK-like homolog) and GlnB proteins are also implicated in the DraT/DraG system, which regulates dinitrogenase reductase activity by covalent modifications [15]. However, Fu et al. [18] verified that *A. amazonense *does not have the DraT/DraG system. Hence, in the near future, the interaction targets of the PII protein in *A. amazonense *should be determined to better understand their roles in the nitrogen metabolism of this microorganism.

### Antibiotic minimum inhibitory concentration

Most DNA manipulation is dependent on the use of vectors containing resistance markers to antibiotics [19,20]. In a previous work using antibiotic susceptibility test discs, Magalhães et al. (1983) [5] showed that *A. amazonense *is sensitive to kanamycin and gentamicin, tolerant to tetracycline, and resistant to penicillin. In this work, we determined the minimum inhibitory concentrations of *A. amazonense *to antibiotics that are normally used to provide a selective pressure for vectors.

The susceptibility of *A. amazonense *to kanamycin and gentamicin was confirmed, since no growth was observed in concentrations of these antibiotics of 0.25 μg/mL; therefore, vectors that contain selection markers for these compounds are appropriate for use.

High concentrations of ampicillin (128 μg/mL) were required for complete growth inhibition, showing that *A. amazonense *is also resistant to this beta-lactam antibiotic.

It is worth noting that the growth of *A. amazonense *was absent in a relatively high concentration of tetracycline (32 μg/mL), indicating that this species is, in fact, resistant to this antibiotic, instead of tolerant, as pointed out by Magalhães et al. [5]. These findings about the latter two antibiotics are relevant because they could be used in counter-selection procedures in conjugation experiments, as there is a variety of *E. coli *strains that are susceptible to them.

### Conjugation

Conjugation mediated by *E. coli *is the standard DNA transfer technique of the *Azospirillum *genus [21]. Therefore, in this work the conjugation ability of *A. amazonense *was evaluated.

Unlike *A. brasilense*, *A. amazonense *cannot grow in LB medium. Furthermore, *E. coli *cannot grow in M79 medium; therefore, the first concern was to establish a medium that provided appropriate growth conditions for the donor and recipient strains. Hence, different medium compositions, containing distinct ratios of M79 and LB media (varying from 1:1 to 9:1), were prepared. The medium mixture of M79:LB at a proportion of 8:2 was the most suitable for culturing both bacteria and it was designated as MLB medium.

Another requisite for the conjugation procedure is to select vectors that contain proper selection markers that are mobilizable and able to replicate inside the receptor cell [19,20]. Therefore, the pHRGFPGUS (pBBR1 replication origin) and the pPZPLACEYFP (pVS1 replication origin) plasmids were tested by tri-parental conjugation. These plasmids are mobilizable broad-host vectors harboring kanamycin resistance markers and fluorescent protein coding genes, which could promptly report achievement of the DNA transfer. The transconjugants exhibited kanamycin resistance and fluorescence. The conjugation frequencies were 3.8 × 10^-8 ^per recipient cell for the pHRGFPGUS vector and 3.8 × 10^-7 ^for the pPZPLACEYFP vector.

Different ratios of recipient to donor and helper strains (1:1:1, 5:1:1, 10:1:1 and 20:1:1) were also tested. The best efficiencies were obtained with the ratios 10:1:1 and 5:1:1; however, no obvious differences between these latter ratios were observed (data not shown).

In conclusion, conjugation is an appropriate method for DNA transfer to *A. amazonense*. Although only tri-parental mating was tested in this work, it is important to mention that bi-parental conjugation could be an alternative test, due to the possibility of increasing the conjugation efficiencies.

### Electrotransformation

Since suitable vectors for *A. amazonense *were defined and since conjugation is a time-consuming procedure, the transformation of *A. amazonense *via electroporation was tested.

The eletrocompetence of the cells is greatly influenced by the growth phase [22]. Therefore, *A. amazonense *cells were harvested at different growth phases to evaluate their effect on electroporation efficiency. Cells from the late-log phase (OD_600 _1) and the stationary phase (OD_600 _2) were not electrocompetent. Electroporation utilizing cells from the early-log growth phase (OD_600 _0.12) generated a significant number of transformants. Therefore, all subsequent tests were performed utilizing cells cultivated at this growth phase.

In the electrocompetent cell preparation, the cells were harvested and washed continuously until the solution had a low-ionic strength. The MgCl_2 _HEPES-sucrose buffer was found to be the most suitable solution for the preparation of *A. amazonense *electrocompetent cells. Although 10% glycerol solution is commonly used for electrocompetent cell preparation in a diverse number of species (including *A. brasilense*), it was not appropriate for *A. amazonense*, as no transformants were obtained when this solution was used.

**Figure 2:**
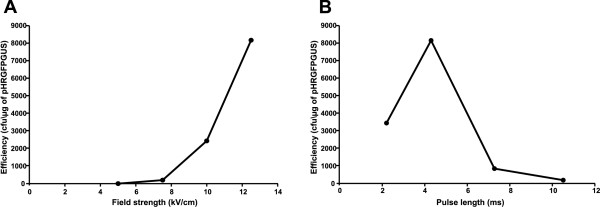
**Electrical parameters tested for the *A. amazonense *electroporation**. A - Effect of electrical field strength on the transformation efficiency of *A. amazonense*. Competent cells were electroporated at the electric field strengths indicated with the pHRGFPGUS vector, with the GenePulser apparatus set at 200 Ω and 25 μF. B - Effect of the pulse length on the transformation efficiency of *A. amazonense*. Competent cells were electroporated with different pulse lengths, using 50 ng of the pHRGFPGUS vector and with the GenePulser apparatus set at 12.5 kV/cm and 25 μF. The pulse lengths 2.2 ms, 4.3 ms, 7.3 ms and 10.5 ms are obtained setting the GenePulser apparatus at 100 Ω, 200 Ω, 400 Ω and 600 Ω, respectively.

Different electroporation parameters were tested. The increase in electrical field strength had a positive effect on electroporation efficiency (Figure 2A). The highest electrical field strength tested was 12.5 kV/cm, and this condition was found to be the most efficient, generating about 8000 transformants/μg of pHRGFPGUS (Figure 2A). The effect of pulse length on electroporation efficiency was also investigated (Figure 2B). A pulse length of 4.3 ms (electroporation apparatus set at 200Ω) was the most efficient. The pulse lengths of 7.3 ms (400 Ω) and 10.5 ms (600 Ω) had a dramatic negative effect on transformation efficiency, where only few transformants were obtained (Figure 2B). These conditions are in agreement with the general parameters of bacterial electroporation [22-24].

In conclusion, the transfer of DNA to *A. amazonense *by means of electroporation was demonstrated. Although the efficiency of electrotransformation was far from desirable, this result is supported by previous works showing that bacteria closely related to *A. amazonense*, such as *A. brasilense *[25], *R. rubrum *[26] and *Magnetospirillum gryphiswaldense *[27], are recalcitrant to electrotransformation. Nonetheless, this technique is an easy and a rapid method of DNA transfer to the cells of *A. amazonense*.

### Site-directed mutagenesis

Site-directed mutagenesis is a fundamental tool for correlating cellular functions with specific regions of the DNA. Therefore, once DNA transfer techniques were established for *A. amazonense*, the next step was to determine a site-directed mutagenesis protocol for this species.

Most of the *A. brasilense *mutants have been generated by the disruptive insertion of an antibiotic resistance cassette into the target gene [14,28-30]. This approach is not recommended when the target gene composes an operon, since the resistance cassette could introduce a polar effect on the expression of the surrounding genes and, consequently, make it difficult to assign a mutant phenotype to the disrupted gene [31].

Therefore, in this work, a site-directed mutagenesis methodology that generates in-frame mutants without the disruptive insertion of a resistance cassette was evaluated. The *glnK *gene was selected for this methodology because subsequent studies of our laboratory will aim to determine the role of the PII proteins in *A. amazonense *metabolism.

**Figure 3:**
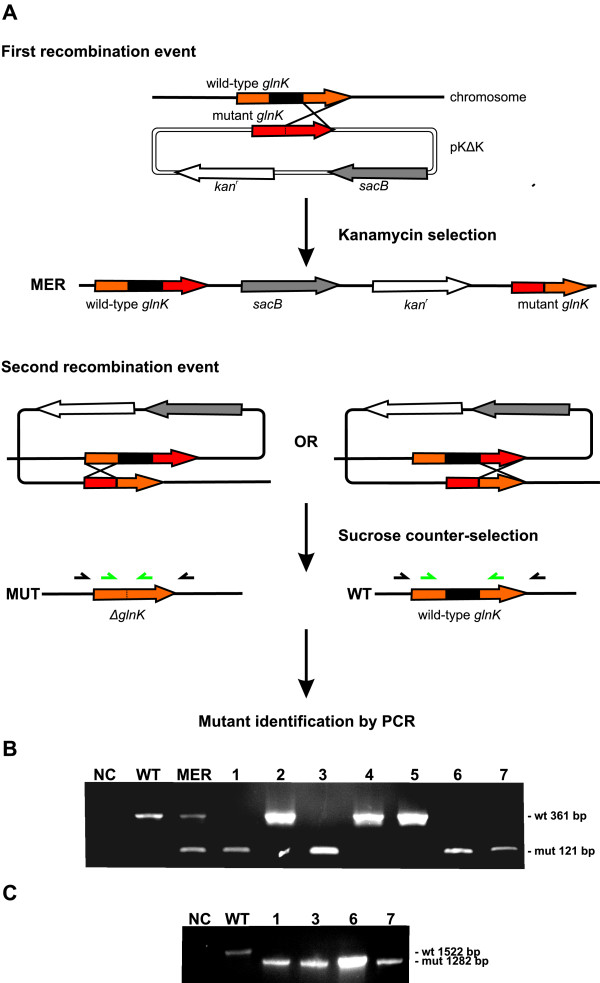
***glnK *gene mutagenesis**. A - Schematic diagram depicting the mutagenesis procedure (modified from Clerico et al., 2007 [42]). The vector pKΔK (pK19MOBSACB derivative) harbors the flanking regions of the *glnK *gene (red). This suicide plasmid was delivered by conjugation to *A. amazonense *and integrated in the target site (orange) by homologous recombination, generating a merodiploid strain (containing both, wild-type and mutant alleles) that was selected by kanamycin since there is a resistance marker (white) present in the vector. The black box represents the region deleted. Subsequently, the merodiploid strain was cultivated and the cells that underwent a second recombination event were selected by sucrose, since the *sacB *marker present in the vector is lethal in the presence of this substance. The kanamycin-sensitive/sucrose resistant colonies were evaluated by PCR. B - Identification of the mutant strains by PCR using primers that flank the deletion site. The primers glnK_NdeI_up and glnK_BamHI_do utilized in this procedure are represented by the small green arrows in Figure 3A. NC - negative control, WT - wild type, MER - merodiploid, numbers - strains tested. C - Verification of the mutant strains by PCR using primers that flank the recombination sites. The primers conf_glnK_up and conf_glnK_do are represented by the small black arrows in Figure 3A. NC - negative control, WT - wild type, numbers - strains tested.

The mutagenesis methodology is depicted in Figure 3A. Firstly, an amplicon containing an in-frame deletion of the *glnK *gene was generated through Crossover PCR, and it was subsequently cloned in the suicide replacement vector pK19MOBSACB, generating the pKΔK plasmid. This vector contains a kanamycin resistance gene (positive selection marker) that allows the selection of bacteria that would have integrated the plasmid into the chromosome. This vector was delivered to *A. amazonense *by means of conjugation (the carbon source utilized was maltose instead of sucrose) and one colony resistant to kanamycin was obtained, suggesting that the integration of the plasmid was successfully accomplished. The *sacB *gene (negative marker selection) of the vector is lethal in the presence of sucrose; therefore, the merodiploid strain (containing both wild-type and mutant alleles) was unable to grow in M79 (containing 10 g/L of sucrose). Subsequently, expecting that a recombination event could replace the wild-type allele, the merodiploid strain was cultured for many generations in M79 containing maltose instead of sucrose. Finally, this culture was plated in M79 containing sucrose to eliminate the bacteria that did not accomplish the second recombination event. Seven sucrose-resistant/kanamycin-sensitive colonies were chosen for PCR evaluation of the substitution of the mutant allele for the wild-type gene. Four colonies presented a band of 121 bp, indicating that the wild-type *glnK *was successfully substituted, whereas three colonies presented the 361 bp band, corresponding to the wild-type allele (Figure 3B). Furthermore, an additional PCR with primers flanking the recombination sites was performed, and it also demonstrated a reduction of the amplicon sizes originated from the *glnK *mutants in relation to the wild type strain (Figure 3C). This latter result demonstrates that recombination occurred in the target site.

Altogether, these results show that an in-frame *glnK *gene mutant strain of *A. amazonense *was successfully generated by this mutagenesis system.

### Reporter gene system

The study of promoters is fundamental to elucidation of the genetic regulatory mechanisms of bacterial species. Up until now, there has been neither a report of heterologous gene expression in *A. amazonense*, nor a reporter system designed for this species. In this work, a reporter system based on expression of the Enhanced Yellow Fluorescent Protein (EYFP) was developed to analyze the regulatory regions of *A. amazonense *genes *in vivo*.

**Figure 4:**
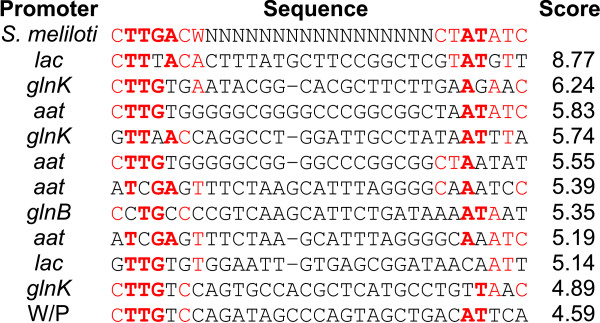
***In silico *sigma 70 promoter analysis**. The upstream sequences of the genes were analyzed by Patser software using an *S. meliloti *sigma 70 factor weight matrix [33]. *aat *- upstream region of the *aat *gene; *glnB *- upstream region of the *glnB *gene; *glnK *- upstream region of the *glnK *gene; *lac *- *lac *promoter; W/P - negative control, 500 bp upstream of the *eyfp *gene of the plasmid pHREYFP. The *S. meliloti *promoter consensus is the first sequence. Nucleotides that match the *S. meliloti *consensus are in red, and those that match the most conserved residues of the *S. meliloti *promoter consensus (relative frequencies above 0.8) are in bold. Gaps were inserted to preserve the alignment at the regions of the promoters.

*In silico *analysis using a *Sinorhizobium meliloti *sigma 70 promoter weight matrix revealed that the genes *aat*, *glnK*, and *glnB *of *A. amazonense *have putative promoter sequences in their upstream regions (Figure 4). In *E. coli*, sigma 70 is considered to be the vegetative sigma factor, as it is responsible for the expression of the majority of genes [32,33]. Therefore, one could expect that these putative *A. amazonense *sigma 70 promoters could act under standard laboratory growth conditions (aerobic environment, 35°C and M79 medium). Consequently, different vectors were constructed to determine the activity of the upstream regulatory sequences of *A. amazonense *genes in the expression of EYFP.

The *lac *promoter was utilized as a positive control since there is a report showing that this promoter has high activity in *A. brasilense *[34]. Two different vectors were constructed with the *lac *promoter, one derived from pPZPLACEYFP (pVS1 replicon) and the other derived from pHRGFPGUS (pBBR1 replicon). The upstream regions of the genes *glnB*, *glnK*, and *aat *were cloned into the pHRGFPGUS derivative.

**Figure 5:**
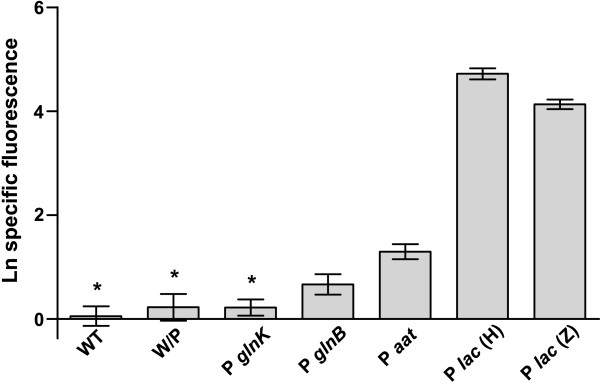
**Analysis of EYFP expression controlled by different *A. amazonense *promoters**. WT- *A. amazonense *without plasmid; W/P - negative control, *A. amazonense *harboring the pHREYFP vector (without promoter); P *glnK *- *A. amazonense *harboring the pHRPKEYFP vector (promoter of *glnK *gene); P *glnB *- *A. amazonense *harboring the pHRPBEYFP vector (promoter of *glnB *gene); P *aat *- *A. amazonense *harboring the pHRAATEYFP vector (promoter of *aat *gene); P *lac *(Z) - *A. amazonense *harboring the pPZPLACEYFP vector (*lac *promoter); P *lac *(H) - *A. amazonense *harboring the pHRLACEYFP vector (*lac *promoter). The error bars represent the confidence interval of 95%, calculated from seven independent experiments (excepting the P *lac *(H), where four experiments were performed). Asterisks indicate activities that do not differ statistically in the Tukey HSD test (P < 0.01).

The *lac *promoter had the best score in the *in silico *analysis from among the promoters detected, and, as expected, the highest fluorescence levels were observed in the *lac *constructions (Figure 5). The difference in the fluorescence levels between the pHRLACEYFP and pPZPLACEYFP transformants could be a product of the difference in the copy number between these vectors.

Although the *in silico *analysis revealed that the *glnK *promoter had a higher score than the *aat *and *glnB *promoters, its *in vivo *activity under the conditions tested did not differ significantly from the negative controls (without promoter and without plasmid) (Figure 5). One of the possible reasons for this is that this gene was repressed under these conditions. The reporter gene analysis also demonstrated that the *aat *and *glnB *promoters were active under the conditions tested, although the *aat *promoter showed a higher activity than the *glnB *promoter.

These observations show that a reporter system based on EYFP can be used for *in vivo *promoter analyses in *A. amazonense*.

## Conclusions

Genetic manipulation is fundamental for taking full advantage of the information generated by DNA sequences [20]. Thus, in the present work, we described a series of tools that could assist genetic studies of the diazotrophic bacteria *A. amazonense*, a microorganism presenting potential for use as an agricultural inoculant.

## Methods

### Bacterial strains, plasmids, and growth conditions

The strains and plasmids utilized in this work are listed in Table 1.

**Table 1:** The strains and plasmids utilized in this work

Bacterial strains	Genotype	Reference	
*Escherichia coli *XL1-Blue	*recA1, endA1, gyrA96, thi-1, hsdR17(rK-, mK+), supE44, relA1, lac, [F', proAB, lacIqZΔM15::Tn10(*tet^r^*)] *	Stratagene	

*Azospirillum amazonense *Y2	wild type	EMBRAPA-RJ	

*Azospirillum amazonense *delK	Y2 derivative, *ΔglnK*	This work	

**Plasmids**	**Relevant characteristics**	**Reference**	**Purpose**

pUC18	cloning vector, amp^r^, *lacZα*	Clontech	cloning procedures

pAAGLNK	pUC18 derivative containing the SalI genomic fragment of the *glnK *region	This work	*glnK *isolation

pGLNBA	pUC18 derivative containing the amplification product generated from the revsf_glmBint and gln_AA_do primers	This work	*glnB*-*glnA *intergenic region and partial *glnA *isolation

pRK2013	ColE1 ori, kan^r^, *mob*, *tra*	[45]	helper plasmid for conjugation experiments

pHRGFPGUS	pBBR1 ori, kan^r^, *mob*, *gfpmut3*	[46]	DNA transfer evaluation, reporter vectors construction

pPZP201BK	pVS1 ori, ColE1 ori, kan^r^, *mob*	[47]	construction of pPZPLACEYFP

pEYFP	pUC ori, *eyfp*, amp^r^	Clontech	contruction of reporter plasmids

pPZPLACEYFP	PvuII-EcoRI fragment from pEYFP (containing the *lac *promoter-*eyfp *gene fusion) cloned into the EcoRV-EcoRI sites of pPZP201BK	This work	DNA transfer evaluation, positive control in the reporter assay

pK19MOBSACB	Integration vector, kan^r^, ColE1 replication origin, *mob*, *sacB*, *lacZ*α	[48]	*glnK *mutagenesis

pKΔK	pK19MOBSACB derivative containing the flanking regions of the *glnK *gene joined by Crossover PCR	This work	*glnK *mutagenesis

pAATEYFP	pEYFP derivative containing the BglII-NcoI fragment of pAAGLNK cloned into its NcoI-BamHI sites	This work	construction of pHRAATEYFP

pPBEYFP	pEYFP derivative containing the intergenic region between the bcr protein superfamily gene and the *glnB *gene cloned into its NcoI-BamHI sites	This work	construction of pHRPBEYFP

pPKEYFP	pEYFP derivative containing the intergenic region between the *ubiH *and the *glnK *genes cloned into its NcoI-BamHI sites	This work	construction of pHRPKEYFP

pHRAATEYFP	HindIII-EcoRI fragment from pAATEYFP (containing the *aat *promoter-*eyfp *gene fusion) joined with the 5.8 kb HindIII-EcoRI fragment of pHRGFPGUS	This work	promoter evaluation

pHRLACEYFP	PvuII-EcoRI fragment from pEYFP (containing the *lac *promoter-*eyfp *gene fusion) joined with the 7.4 kb EcoRV-EcoRI fragment of pHRGFPGUS	This work	promoter evaluation

pHRPBEYFP	HindIII-EcoRI fragment from pPBEYFP (containing the glnB promoter-eyfp gene fusion) joined with the 5.8 kb HindIII-EcoRI fragment of pHRGFPGUS	This work	promoter evaluation

pHRPKEYFP	HindIII-EcoRI fragment from pPKEYFP (containing the *glnK *promoter-*eyfp *gene fusion) joined with the 5.8 kb HindIII-EcoRI fragment of pHRGFPGUS	This work	promoter evaluation

pHREYFP	HindIII-EcoRI fragment from pEYFP (containing the *eyfp *gene) joined with the 5.8 kb HindIII-EcoRI fragment of pHRGFPGUS	This work	promoter evaluation

*Azospirillum amazonense *was cultured in M79 medium (10 g/L of sucrose as the carbon source, 0.1 g/L of K_2_HPO_4_, 0.4 g/L of KH_2_PO_4_, 0.2 g/L of MgCl_2_.7H_2_O, 0.1 g/L of NaCl, 0.4 g/L of yeast extract, pH 6.5) [35] at 35°C (unless stated otherwise). The M79 agar plates contained 2.5 mg/L of Bromothymol Blue. *Escherichia coli *XL1-Blue was cultured in LB medium at 37°C [36].

### DNA techniques

Standard DNA techniques such as PCR, plasmid extraction, DNA restriction and modification, gel electrophoresis, and *E. coli *transformation were carried out as described in Sambrook and Russell (2001) [36]. The total DNA extraction of *A. amazonense *was performed as described by Wilson (1997) [37]. The primers used for PCR are listed in Table 2. All of the restriction and modification enzymes utilized in this work were purchased from New England Biolabs. The Taq DNA polymerase was provided by CenBiot Enzimas (Centro de Biotecnologia, UFRGS).

**Table 2:** Primers utilized in this work

Primers	Sequence	Annealing temperature	Amplicon length (bp)	Purpose
glnB_sfint	CGCCGCGATACAGCTCGGTATG	57°C	2108	*glnB *region isolation

revsf_glnBint	GATGGACGATCAGTTGGTCGA	57°C	2108	*glnB *region isolation

glnA_aa_do	ACGGTCGGCACTTCCTTCAG	53°C	1621	*glnB *region isolation

pglnB_up_BamHI	CGGGATCCTCGTCGAAGCTGAAGGTCAT	55°C	727	*glnB *promoter amplification

pglnB_do_NcoI	GATCTTTTCCATGGCTTACGGC	55°C	727	*glnB *promoter amplification

pglnK_up_BamHI	CGGGATCCTTGTCCAGTGCCACGCTCAT	55°C	328	*glnK *promoter amplification

pglnK_do_NcoI	CACGAGCTCCATGGGTAGTCC	55°C	328	*glnK *promoter amplification

Kglndel_A _EcoRI	ATGAATTCCAATGCACAGGGTGCGTA	55°C	574 (AB) and 1111 (AD)	*glnK *mutagenesis

Kglndel_B	**CCCATCCACTAAACTTAAACA**CGCCACCACGAGCTTCAT	55°C	574	*glnK *mutagenesis

Kglndel_C	**TGTTTAAGTTTAGTGGATGGG**ATGACCATCGCCGACGCG	55°C	558	*glnK *mutagenesis

Kglndel_D_BamHI	CGGGATCCCGATGGTGGGCGGATATTTG	55°C	558 (CD) and 1111 (AD)	*glnK *mutagenesis

glnK_NdeI_up	GGACTACATATGAAGCTCGTGGTG	60°C	361 (wt) or 121 (mut)	*glnK *mutagenesis verification

glnK_BamHI_do	CGTCACGGGATCCTCATAAGGC	60°C	361 (wt) or 121 (mut)	*glnK *mutagenesis verification

conf_glnK_up	GCCCCCTCCAGGATCTTC	55°C	1522 (wt) or 1282 (mut)	*glnK *mutagenesis verification

conf_glnK_do	GGGTAAAATGCCCTTGTCCA	55°C	1522 (wt) or 1282 (mut)	*glnK *mutagenesis verification

Underline - restriction sites; Bold - sequence tag; wt - wild-type; mut - mutant; AB - amplification using the primers KglndelA_EcoRI and KglndelB; AD - amplification using the primers Kglndel_A_EcoRI and Kglndel_D_BamHI; CD - amplification using the primers KglndelC and Kglndel_D_BamHI

### Isolation of *glnB *and *glnK *genes from *A. amazonense*

The genomic library enriched with 2-3 kb SalI DNA fragments was constructed as follows: the genomic DNA was digested with SalI and subsequently separated in agarose gel by electrophoresis. The 2-3 kb fragments were excised from the agarose gel and purified. Finally, these fragments were cloned in the pUC18 plasmid. This genomic library was partially sequenced and the *glnK *gene was identified using BLAST searches.

Inverse PCR for *glnB *isolation was performed according to Sambrook and Russell (2001) [36]. *Azospirillum amazonense *genomic DNA was digested with SalI and subsequently circularized. The PCR was performed with the glnB_sfint and revsf_glnBint primers (Table 2) and the circularized SalI DNA as a template. The 5' portion of the *glnA *gene was isolated by PCR with the revsf_glnBint and glnA_aa_do primers (Table 2) and the genomic DNA as a template.

The DNA sequencing was performed using a MEGABACE automated platform (Centro de Biotecnologia, UFRGS). Sequences were assembled using the Staden software package [39]. Gene annotation was carried out by Artemis software version 12.0 [40] along with BLAST software using the NCBI database http://blast.ncbi.nlm.nih.gov/Blast.cgi. Both sequences were deposited in the NCBI nucleotide database under the following access numbers: *glnB *region [GenBank:HM161849] and *glnK *region [GenBank:HM161850].

### Antibiotic minimum inhibitory concentration test

The minimum inhibitory concentration of *A. amazonense *to the antibiotics (gentamicin, kanamycin, tetracycline, and ampicillin) was basically evaluated as described in Andrews (2001) [41]. The antibiotics were serially diluted in 1 mL of M79 medium at concentrations from 256 μg/mL to 0.5 μg/mL. An overnight culture of *A. amazonense *was diluted to 4 × 10^4 ^cells/mL. One milliliter of this dilution was added to one milliliter of M79 medium containing the appropriate antibiotic concentration. The cells were cultivated in a rotary shaker at 150 rpm for 40 h at 35°C.

### Conjugation

Conjugation was basically carried out as described by Clerico et al. (2007) [42]. However, some modifications were made as follows: overnight cultures of *A. amazonense *Y2 (receptor), *E. coli *XL1-Blue containing the plasmid pRK2013 (helper), and *E. coli *XL1-Blue containing the appropriate plasmid (donor) were used. Approximately 1 mL of the *A. amazonense *culture with an OD_600 _= 2 (1.3 × 10^9 ^cfu/ml) was mixed with 1 mL of each helper and donor cultures with an OD_600 _= 0.2 (2 × 10^8 ^cfu/mL) (ratio 10:1:1), unless stated otherwise. This mixture was harvested by centrifugation at 6000 *g *for 2 min and then resuspended in 100 μL of MLB medium (LB and M79 mixture at a proportion of 8:2), and this volume was then spotted onto MLB agar and incubated for 20 h at 35°C. Following this, the cell mass was resuspended in 200 μL of M79 medium and plated on M79 medium containing the appropriate antibiotic.

### Electroporation

The preparation of cells was based on the protocol described by Schultheiss and Schüler (2003) [27]. A 3 mL overnight culture of *A. amazonense *was inoculated in 250 mL of M79 and the cells were cultivated to an OD_600 _of ~0.12 (early-log growth phase), unless stated otherwise. From this point, all manipulations were conducted on ice. The cells were incubated in ice for 30 min and then harvested by centrifugation at 5000 *g *for 20 min at 10°C. The cells were resuspended in 100 mL of electroporation buffer (pH 6.5 HEPES 1 mM, MgCl_2 _1 mM, and sucrose 200 mM) and again harvested by centrifugation (20 min at 5000 *g*). Subsequently, the cells were resuspended in 40 mL of electroporation buffer and again harvested by centrifugation. At the end, the cells were resuspended in 250 μL of electroporation buffer (final concentration of ~10^10 ^cfu/mL), distributed in aliquots of 40 μL, and frozen in liquid nitrogen. Cell electroporation was carried out as follows: the 40 μL aliquot was mixed with 50 ng of the pHRGFPGUS vector and electroporated through a Gene Pulser apparatus (Bio-Rad Laboratories Inc.) with 12.5 kV/cm, 25 μF and 200 Ω, unless stated otherwise. After electrical discharge, the cells were resuspended in 500 μL of M79 medium and incubated at 35°C for 3 h in a rotary shaker at 150 rpm. Subsequently, the cells were plated on solid M79 medium containing 20 μg/mL of kanamycin and incubated for 2 days at 35°C.

### Gene mutagenesis

Site-directed mutagenesis was based on a protocol described by Eggeling and Reyes (2005) [43]. In summary, the flanking regions of the *glnK *gene were amplified using the primers KglndelA_EcoRI/KglndelB and KglndelC/KglndelD_BamHI (Table 2). These amplification products were joined by Crossover PCR [31] using the primers KglndelA_EcoRI/KglndelD_BamHI (Table 2) and cloned in pK19MOBSACB digested with EcoRI and BamHI, generating the plasmid pKΔK (Table 1). Subsequently, the vector pKΔK was transferred to *A. amazonense *by conjugation, as previously described, except that the medium utilized was MLB containing maltose instead of sucrose (10 g/L) and ampicillin (100 μg/mL) for the counter-selection of *E. coli*. A kanamycin-resistant colony was isolated and cultured overnight in 3 mL of M79 (containing 10 g/L of maltose instead of sucrose). The culture was serially diluted and plated on M79 medium (containing 10 g/L of sucrose). Fifty sucrose-resistant colonies were replica plated onto both kanamycin-containing and pure M79 agar plates. Seven kanamycin-sensitive/sucrose-resistant colonies were submitted to Touchdown-PCR to identify those that had replaced the wild-type *glnK *gene with the mutant allele. The Touchdown-PCR was performed using the primers glnK_NdeI_up and glnK_BamHI_do (Table 2) under the following conditions: an initial denaturing step of 94°C for 5 min; 15 cycles of 94°C for 30 s, 60°C-56°C for 30 s (for each three cycles one degree was decreased), and 72°C for 30 s; 15 cycles at 94°C for 30 s, 55°C for 30 s, and 72°C for 30 s. The PCR utilizing the primers Conf_glnK_up and Conf_glnK_do (Table 2), which flank the recombination sites of the *glnK *region, was carried out in the same way as standard PCR procedures [36].

### Gene reporter system

The upstream sequences of the genes utilized in this work were analyzed by Patser (available on the RSAT webserver) [44] with an *S. meliloti *sigma 70 factor weight matrix [33].

A series of reporter vectors was developed to evaluate the activity of different promoters (Table 1). The upstream regions of the *glnB *and *glnK *genes were amplified utilizing the primers listed in Table 2. Subsequently, these amplicons were cloned into the pEYFP vector at the NcoI and BamHI sites, generating pPBEYFP and pPKEYFP plasmids, respectively. After evaluation of the integrity of these amplicons by automated sequencing, the HindIII-EcoRI fragment, containing the promoter-*eyfp *fusion, was transferred to the HindIII-EcoRI fragment of pHRGFPGUS, which contains the replication origin, the mobilization site, and the kanamycin resistance marker, generating the pHRPBEYFP and pHRPKEYFP plasmids, respectively.

The pHRAATEYFP plasmid was constructed in the following way: the NcoI-BglII fragment of pAAGLNK, containing the upstream region of the *aat *gene, was transferred to pEYFP, generating the plasmid pAATEYFP. The HindIII-EcoRI fragment from this plasmid was transferred to the HindIII-EcoRI fragment of pHRGFPGUS, generating pHRAATEYFP.

The negative control plasmid, which did not contain a promoter, was constructed as follows: the NcoI-BamHI fragment of pEYFP was transferred to the HindIII-EcoRI fragment of pHRGFPGUS, forming the plasmid pHREYFP.

The positive control plasmid pHRLACEYFP is a fusion of the major EcoRI-EcoRV fragment of pHRGFPGUS with the PvuII-EcoRI fragment of pEYFP.

All of the plasmids were transferred to *A. amazonense *by tri-parental mating or electroporation. The promoter activity assay was basically performed as described in MacLellan et al. (2006) [33]. *Azospirillum amazonense *containing the reporter vectors was cultivated in M79 medium overnight in a rotary shaker at 35°C. The cells were washed in sterile saline solution (0.85% NaCl) and resuspended in this same solution to an OD_600 _of between 0.06-0.39. Two hundred microlitres of the cell suspensions were deposited on black microtiter plates and fluorescence was measured with an excitation wavelength of 488 nm and an emission wavelength of 527 nm. The optical densities of the cell suspensions were measured at 600 nm on clear microtiter plates. Specific fluorescence was obtained by dividing the fluorescence by the optical density. Statistical analysis was performed using SAS JMP8 software: the specific fluorescence data was subjected to the natural logarithm to homogenize the variances (tested by Levene's test) and subsequently submitted for ANOVA/Tukey HSD tests (P < 0.01).

## Authors' contributions

FHS conceived, coordinated and carried out the research study, drafted the manuscript, and created the illustrations and the tables. DSA performed the antibiotic minimum inhibitory concentration tests and helped with the electroporation procedures. DBT helped to isolate the *glnB *gene, designed some primers, and revised the manuscript. SSW helped with the reporter assays, and revised the manuscript. ISS conceived and coordinated the study, and revised the manuscript. All authors read and approved the final manuscript.
